# A balancing act: ERFVII feedback loops finetune the flooding stress response

**DOI:** 10.1093/plphys/kiad609

**Published:** 2023-11-14

**Authors:** Aida Maric

**Affiliations:** Assistant Features Editor, Plant Physiology, American Society of Plant Biologists; CIBSS—Centre for Integrative Biological Signalling Studies, University of Freiburg, Habsburgerstraße 49, 79104, Freiburg, Germany; Plant Environmental Signalling and Development, Institute of Biology III, University of Freiburg, Schänzlestraße 1, 79104 Freiburg, Germany

Plants are masters of environmental adaptation. Flooding is one of the environmental stresses that plants face ever more frequently as a consequence of climate change. The first signal of submergence stress is the accumulation of gaseous phytohormone ethylene (Fig. A). Ethylene mediates the adaptation to submergence stress by regulating a battery of Ethylene Response Factors (ERFs) at the transcriptional, translational, and post-translational level (Maric and Hartman 2022). A specific family of ERFs, the ERFVIIs, are part of a cellular oxygen (O_2_) and nitric oxide (NO) sensing mechanism because their stability is directly controlled by the concentration of these gases (Gibbs et al. 2011; Licausi et al. 2011; Gibbs et al. 2014). Indeed, ERFVIIs stabilize during O_2_ deprivation (hypoxia) and NO depletion (Gibbs et al. 2014; Holdsworth and Gibbs 2020). Ethylene can further promote ERFVII stability in Arabidopsis through the fast induction of a plant hemoglobin, *PHYTOGLOBIN 1* (*PGB1*) (Hartman et al. 2019). PGB1 is a NO scavenger that can limit the NO-dependent proteolysis of ERFVIIs (Hartman et al. 2019). Consequently, in response to the flooding signals ethylene and hypoxia, the ERFVIIs quickly accumulate and start a signaling pathway increasing plant tolerance to flooding.

**Figure.**
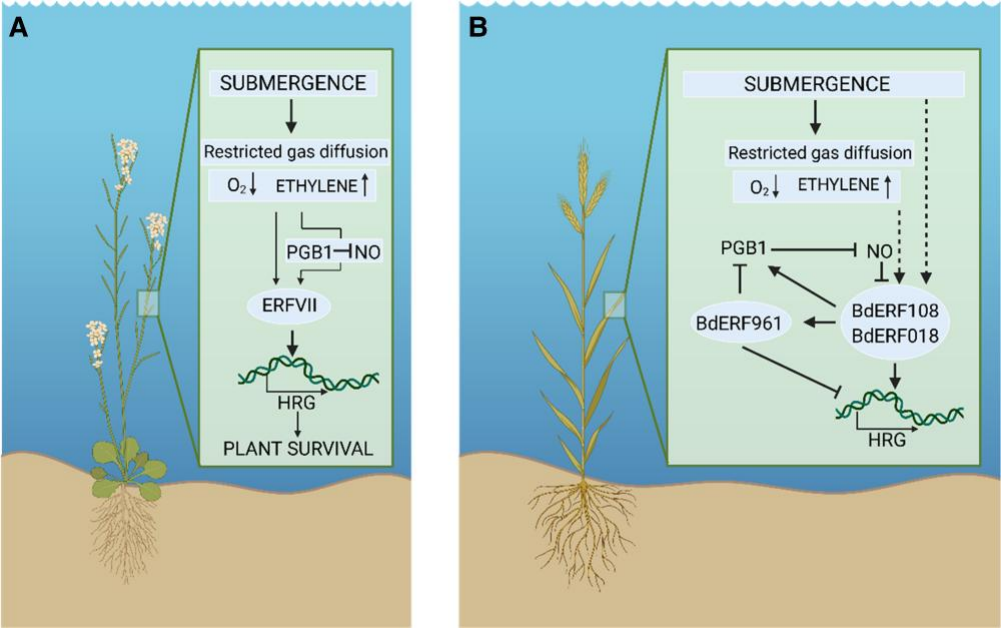
Plant submergence response. **A)** In the model plant Arabidopsis, depleting levels of O_2_ accumulation upon submergence mediate the stabilization of ERFVIIs. Furthermore, the accumulation of ethylene and of NO scavenger PGB1 further stabilize the ERFVIIs. ERFVIIs activate the transcription of hypoxia response genes (HRGs), leading to plant survival. **B)** Upon submergence of cereal model plant *Bracyhpodium distachyon*, 2 BdERFVIIs—BdERF108 and BdERF018—activate the expression of HRGs, including *PGB1*. PGB1 depletes the levels of NO, preventing the degradation of BdERF108 and BdERF018. However, BdERF108 and BdERF018 also activate expression of BdERF961. BdERF961 represses the HRGs and PGB1, thereby stabilizing the NO and promoting the degradation of BdERFVIIs. Arrowheads and flatheads represent activation and inhibition, respectively. Dashed lines represent hypotheses. Figure adapted from Hsiao et al. (2023). Created with BioRender.com.

ERFVIIs are a gene family widely conserved across plant species. However, they have developed some species-specific characteristics. Rice SUB1A is one of the most famous ERFVIIs because overexpression of SUBA1A can enhance flooding tolerance in submergence-intolerant rice cultivars (Bailey-Serres et al. 2010; Xu et al. 2006). Other well-known rice ERFVIIs, SNORKEL1 (SK1) and SK2, regulate flooding survival responses but through entirely different flooding escape mechanisms (Hattori et al. 2009). Moreover, not all ERFVIIs are controlled by O_2_ and ethylene, and some do not seem to play a role in flooding acclimation at all.

In a newly published *Plant Physiology* article, Hsiao et al. (2023) investigated the battery of ERFVIIs in *Bracyhpodium distachyon*, a model cereal species. The authors identified a group of 3 ERFVIIs in Bracyhpodium that regulate the submergence response. BdERF108 and BdERF018 activate the submergence response by inducing the expression of *PGB1* (Fig. B). Simultaneously, BdERF108 and BdERF018 activate the expression of the third member of the group, *BdERF961* (Fig. B). In turn, BdERF961 acts as a negative regulator of the submergence response by repressing the expression of various hypoxia response genes and *PGB1* (Fig. B). This work offers the first example of an ERFVII, BdERF961, that negatively regulates the response to submergence instead of promoting it.

The authors investigated whether this novel negative regulation of the hypoxia response exhibited by BdERF1 is conserved in rice. They identified the rice ortholog of BdERF961 as OsERF67. Unlike BdERF961, OsERF67 serves as a positive regulator of the submergence stress response, indicating that the function is not conserved between Brachypodium and rice. In the future, it would be interesting to find out how these transcription factors have evolved to become positive and negative regulators of the flooding stress response. What is the molecular mechanism of specialization of these transcription factors? And what are the mechanisms behind the species-specific evolution of such an intricate submergence stress response?

This new work from Hsiao et al. (2023) characterized a molecular feedback loop between members of the ERFVII family in Brachypodium, which represents an elegant way of self-attenuating response to the submergence stress. This opens many questions on how these interactions tailor the molecular output in different contexts, such as prolonged submergence, reoxygenation, and recurrent flooding (stress memory). Can negative regulators be removed to promote flooding tolerance, and what is the cost of this removal for yield?

Moreover, this work offers one of the rare insights into the maintenance of ERFVII stability through plant PGB-dependent NO scavenging, which itself is under the control of hypoxia through the ERFVIIs and ethylene. How do the dynamics of ERFVII activity and its feedback loops regulate submergence stress responses in spatiotemporal detail to come to a tailored response? Maintaining a balance between growth and stress response is the eternal dilemma for plants facing stress. This work sets us on an important pathway of understanding how different species maintain that balance in the face of the evermore present submergence stress.

## Dive Curated Terms

The following phenotypic, genotypic, and functional terms are of significance to the work described in this paper:

ethylene Gramene: Hormone
ethylene Araport: Hormone

## References

[kiad609-B1] Bailey-Serres J , Fukao T, Ronald P, Ismail A, Heuer S, Mackill D. Submergence tolerant rice: SUB1’s journey from landrace to modern cultivar. Rice. 2010:3(2–3):138–147. 10.1007/s12284-010-9048-5

[kiad609-B2] Gibbs DJ , Lee SC, Isa NM, Gramuglia S, Fukao T, Bassel GW, Correia CS, Corbineau F, Theodoulou FL, Bailey-Serres J, et al Homeostatic response to hypoxia is regulated by the N-end rule pathway in plants. Nature. 2011:479(7373):415–418. 10.1038/nature10534 22020279 PMC3223408

[kiad609-B3] Gibbs DJ , Md Isa N, Movahedi M, Lozano-Juste J, Mendiondo GM, Berckhan S, Marín-de la Rosa N, Vicente Conde J, Sousa Correia C, Pearce SP, et al Nitric oxide sensing in plants is mediated by proteolytic control of group VII ERF transcription factors. Mol Cell. 2014:53(3):369–379. 10.1016/j.molcel.2013.12.020 24462115 PMC3969242

[kiad609-B4] Hartman S , Liu Z, van Veen H, Vicente J, Reinen E, Martopawiro S, Zhang H, van Dongen N, Bosman F, Bassel GW, et al Ethylene-mediated nitric oxide depletion pre-adapts plants to hypoxia stress. Nat Commun. 2019:10(1):4020. 10.1038/s41467-019-12045-4 31488841 PMC6728379

[kiad609-B5] Hattori Y , Nagai K, Furukawa S, Song XJ, Kawano R, Sakakibara H, Wu J, Matsumoto T, Yoshimura A, Kitano H, et al The ethylene response factors SNORKEL1 and SNORKEL2 allow rice to adapt to deep water. Nature. 2009:460(7258):1026–1030. 10.1038/nature08258 19693083

[kiad609-B6] Holdsworth MJ , Gibbs DJ. Comparative biology of oxygen sensing in plants and animals. Curr Biol. 2020:30(8):R362–R369. 10.1016/j.cub.2020.03.021 32315638

[kiad609-B7] Hsiao P-Y , Zeng C-Y, Shih M-C. Group VII ethylene response factors forming distinct regulatory loops mediate submergence responses. Plant Physiol. 2024:194(3):1745–1763. 10.1093/plphys/kiad547 PMC10904320 37837603

[kiad609-B8] Licausi F , Kosmacz M, Weits DA, Giuntoli B, Giorgi FM, Voesenek LA, Perata P, van Dongen JT. Oxygen sensing in plants is mediated by an N-end rule pathway for protein destabilization. Nature. 2011:479(7373):419–422. 10.1038/nature10536 22020282

[kiad609-B9] Maric A , Hartman S. Ethylene controls translational gatekeeping to overcome flooding stress in plants. EMBO J. 2022:41(19):e112282. 10.15252/embj.2022112282 35975893 PMC9531296

[kiad609-B10] Xu K , Xu X, Fukao T, Canlas P, Maghirang-Rodriguez R, Heuer S, Ismail AM, Bailey-Serres J, Ronald PC, Mackill DJ. Sub1A is an ethylene responsive-factor-like gene that confers submergence tolerance to rice. Nature. 2006:442(7103):705–708. 10.1038/nature04920 16900200

